# Reducing Alcohol Use During Pregnancy Via Health Counseling by Midwives and Internet-Based Computer-Tailored Feedback: A Cluster Randomized Trial

**DOI:** 10.2196/jmir.3493

**Published:** 2014-12-05

**Authors:** Nickie Y van der Wulp, Ciska Hoving, Kim Eijmael, Math JJM Candel, Wim van Dalen, Hein De Vries

**Affiliations:** ^1^Dutch Institute for Alcohol Policy STAPUtrechtNetherlands; ^2^Care and Public Health Research InstituteMaastricht UniversityMaastrichtNetherlands; ^3^Department of Health PromotionMaastricht UniversityMaastrichtNetherlands; ^4^Department of Methodology and StatisticsMaastricht UniversityMaastrichtNetherlands

**Keywords:** alcohol drinking, pregnancy, counseling, telemedicine, midwifery

## Abstract

**Background:**

Effective interventions are needed to reduce neurobehavioral impairments in children due to maternal alcohol use during pregnancy. Currently, health-counseling interventions have shown inconsistent results to reduce prenatal alcohol use. Thus, more research using health counseling is needed to gain more knowledge about the effectiveness of this type of intervention on reducing alcohol use during pregnancy. An alternative and promising strategy is computer tailoring. However, to date, no study has shown the effectiveness of this intervention mode.

**Objective:**

The aim was to test the effectiveness of health counseling and computer tailoring on stopping and reducing maternal alcohol use during pregnancy in a Dutch sample of pregnant women using alcohol.

**Methods:**

A total of 60 Dutch midwifery practices, randomly assigned to 1 of 3 conditions, recruited 135 health counseling, 116 computer tailoring, and 142 usual care respondents from February to September 2011. Health-counseling respondents received counseling from their midwife according to a health-counseling protocol, which consisted of 7 steps addressed in 3 feedback sessions. Computer-tailoring respondents received usual care from their midwife and 3 computer-tailored feedback letters via the Internet. Usual care respondents received routine alcohol care from their midwife. After 3 and 6 months, we assessed the effect of the interventions on alcohol use.

**Results:**

Multilevel multiple logistic regression analyses showed that computer-tailoring respondents stopped using alcohol more often compared to usual care respondents 6 months after baseline (53/68, 78% vs 51/93, 55%; *P*=.04). Multilevel multiple linear regression analyses showed that computer-tailoring respondents (mean 0.35, SD 0.31 units per week) with average (*P*=.007) or lower (*P*<.001) alcohol use before pregnancy or with average (*P*=.03) or lower (*P*=.002) social support more strongly reduced their alcohol use 6 months after baseline compared to usual care respondents (mean 0.48, SD 0.54 units per week). Six months after baseline, 72% (62/86) of the health-counseling respondents had stopped using alcohol. This 17% difference with the usual care group was not significant.

**Conclusions:**

This is the first study showing that computer tailoring can be effective to reduce alcohol use during pregnancy; health counseling did not effectively reduce alcohol use. Future researchers developing a health-counseling intervention to reduce alcohol use during pregnancy are recommended to invest more in recruitment of pregnant women and implementation by health care providers. Because pregnant women are reluctant to disclose their alcohol use to health professionals and computer tailoring preserves a person’s anonymity, this effective computer-tailoring intervention is recommended as an attractive intervention for pregnant women using alcohol.

**Trial Registration:**

Dutch Trial Register NTR 2058; http://www.trialregister.nl/trialreg/admin/rctview.asp?TC=2058 (Archived by WebCite at http://www.webcitation.org/6NpT1oHol)

## Introduction

Alcohol use in pregnancy is a leading preventable cause of intellectual disability in children [1]. Due to accumulating evidence that even low levels of prenatal alcohol exposure can cause adverse neurobehavioral effects in children [2], many Western countries, such as the United States, Australia, and the Netherlands, officially recommend that pregnant women completely abstain from alcohol [3-5]. Nevertheless, more than 20% of pregnant women worldwide consume alcohol [6], with estimations accumulating to 35% to 50% in the Netherlands [4]. Effective interventions are needed to reduce the number of pregnant women who endanger the health of their fetuses by using alcohol in pregnancy.

Various interventions to reduce prenatal alcohol use have been described in reviews [7-9]; however, only 5 studies used a randomized controlled trial to test intervention effectiveness (ie, [10-14]). All these interventions applied health counseling; pregnant women were screened for alcohol use and participated in motivational interviews conducted by health professionals (eg, [15]). These studies suggest that health-counseling interventions may result in increased abstinence and a reduction in prenatal alcohol consumption. However, because of the inconsistency of the results, the paucity of studies, the relatively low number of total respondents, the high risk of bias of the studies due to lack of information on allocation concealment, and the complexities of interventions, many uncertainties remain about the most optimal conditions of these interventions [9].

An alternative and promising strategy is computer tailoring, an intervention in which advice is not delivered face-to-face, but via a computer [16]. The content of this advice is based on the answers of respondents to questions and is generated by a computer program. Consequently, the feedback is adapted to the specific characteristics of a particular individual, yielding the potential to provide messages highly tailored to the individuals’ situation [17]. Computer-tailored messages have been shown to attract and keep an individual’s attention [16,18] more than generic advice, resulting in a more thorough processing of information [19]. Computer tailoring has proven to be effective in changing different health-related behaviors [20,21], such as smoking [22], vegetable and fruit intake [23], and alcohol use [24,25]. To our knowledge, only 1 computer-tailored intervention for alcohol use in pregnancy has been developed. Tzilos and colleagues [26] found that users liked the program and appreciated the ease of use. Nevertheless, they did not find any significant difference in the reduction of alcohol use compared to an assessment-only condition, perhaps because their 1-month follow-up was simply too soon to find beneficial effects of the computer tailoring or because their phone-based follow-up led to a social desirability bias concealing real decreases in drinking. Thus, it has not been shown that computer tailoring can be effective in reducing prenatal alcohol use.

The goal of this study was to test the effectiveness of 2 different brief interventions to reduce prenatal alcohol use, a health counseling and a computer-tailored intervention, in comparison with usual care. In agreement with several national recommendations [3-5], our primary focus for the development of the interventions was that pregnant women who used alcohol in the beginning of their pregnancy stopped their alcohol use after having received an intervention. Thus, our first hypothesis was that women receiving health counseling or computer tailoring were more likely to stop using alcohol in pregnancy compared to women receiving usual care. However, for the pregnant women unwilling or unable to completely stop their alcohol use, we aimed at reducing their alcohol use because research has shown that the risk and severity of the effects of prenatal alcohol use are dose-related [27]. Consequently, our second hypothesis is that when women continued their alcohol use, those receiving health counseling or computer tailoring were more likely to reduce their alcohol use compared to those receiving usual care.

## Methods

### Ethical Approval and Registration

The study was approved by the Medical Ethics Committee of Maastricht University and the University Hospital Maastricht (MEC 09-3-070) and is registered with the Dutch Trial Register (NTR2058).

### Sample

A sample size analysis, with power=.80, alpha=.05, intraclass correlation coefficient (ICC) of 0.01 (reported in a previous study as the median ICC for cluster-based studies in primary care [28]), an estimated quit rate of 40% in each of the experimental conditions versus 20% in the control condition, and the estimated inclusion of 30 midwifery practices, revealed that 9 respondents per practice were needed. Estimating 10% attrition over the trial period, we aimed to include 300 respondents at baseline. The estimated quit rate and attrition were based on a previous Dutch study on smoking cessation during pregnancy [29].

Eligibility criteria were ability to understand Dutch, aged 18 years or older, pregnant for a maximum of 12 weeks (because respondents received follow-up questionnaires until 6 months after baseline), and having drunk alcohol since knowing to be pregnant.

### Procedures

Respondents were recruited from February to September 2011. Recruitment letters were sent to all midwifery practices in the Netherlands (N=540). Participating practices were randomly assigned to 1 of the 3 conditions (health counseling, computer tailoring, or usual care) by a computer software randomization device to avoid contamination. The practices informed their clients about the study by email or phone. When pregnant women agreed to participate, they were asked to visit the study website before their initial consultation. They could do this where and whenever they had access to the Internet.

During the recruitment period, it appeared that the inclusion of 9 respondents per practice would be too time-consuming. We decided to enroll 60 midwifery practices in total, expecting to recruit 4-5 respondents from each practice.

The study website included the baseline questionnaire (T0). Respondents could choose their own username and password and had to report their email address when signing up for the study. This way we could easily remove respondents with multiple identities from further analyses. Before providing informed consent, pregnant women were informed about the 3 study conditions and received information about the objectives of the study, the randomization procedure, and the incentive of a €10 voucher when respondents completed all questionnaires and institutional affiliations (“This research is conducted by the Dutch Institute for Alcohol Policy [STAP] and Maastricht University”). After providing online informed consent, eligible women gained access to the baseline questionnaire. Blinding of respondents was not possible because they had to take notice of whether they did or did not receive additional counseling from their midwife (after the baseline questionnaire) or tailored feedback via the computer (during the baseline questionnaire).

At both 3 and 6 months after the baseline questionnaire, all participants received an invitation by email (followed by 2 reminders after 2 and 4 weeks) for the first follow-up questionnaire (T1 and T2, respectively). Nonrespondents after 2 reminders were contacted by telephone to collect their data.

### Interventions

#### Overview

The health counseling and computer-tailoring interventions were both based on the I-Change model [30], a theoretical model incorporating concepts from several social cognitive models, such as the Transtheoretical model [31] and the Theory of Planned Behavior [32]. The I-Change model distinguishes 3 phases of health behavior change (awareness, motivation, and action) and has been used successfully for developing various health promoting interventions, such as prenatal smoking cessation [29], smoking cessation [22,33,34], and increasing vegetable and fruit intake and physical activity [21].

#### Health Counseling

Midwives in the health-counseling condition received a brief manual explaining the health-counseling protocol and an intervention card with questions for the clients. On this intervention card, midwives could record the dates of the health counseling sessions and the clients’ answers to the midwife’s questions. Midwives received 3 hours of training on how to provide the health counseling. This training was given either at the research institute of the first author or at the practice of the participating midwife. The materials and training were based on earlier work on tobacco and pregnancy [29].

The health-counseling protocol consisted of 7 steps which were addressed in 3 feedback sessions. Feedback session 1, approximately 2 weeks after baseline assessment, consisted of 5 steps taking approximately 10 minutes of the initial consultation (Feedback 1-health counseling). In step 1, the midwife assessed the amount and frequency of alcohol use of the pregnant woman before and during pregnancy, of her partner during pregnancy, and the pregnant woman’s motivation to stop drinking alcohol. In step 2, women strongly motivated to stop alcohol consumption during pregnancy were prompted to state the advantages of abstinence. Moderately or not motivated women were asked to report on their perceived disadvantages of drinking during pregnancy. The midwife then advised them to stop drinking alcohol. In step 3, the barriers for successful abstinence and the mobilization of social support were discussed. In step 4, a self-help guide, adapted from an intervention on smoking in pregnancy [29], and relevant websites were mentioned. The midwife stimulated the pregnant woman to develop action plans for abstinence and coping with problems they might encounter when trying not to drink alcohol. If appropriate, access to alcohol addiction services was discussed. In step 5, women were asked to set a date for stopping their alcohol use (goal setting). Feedback session 2, approximately 8 weeks after baseline, consisted of step 6, which was addressed in approximately 1 minute (Feedback 2-health counseling). In this step, midwives again assessed the alcohol use of the pregnant women and asked her if she needed additional support for not drinking alcohol. Feedback session 3, approximately 14 weeks after baseline, consisted of step 7, which was also addressed in approximately 1 minute (Feedback 3-health counseling). In this step, midwives discussed alcohol use and its implications for breastfeeding.

#### Computer Tailoring

**Figure 1:**
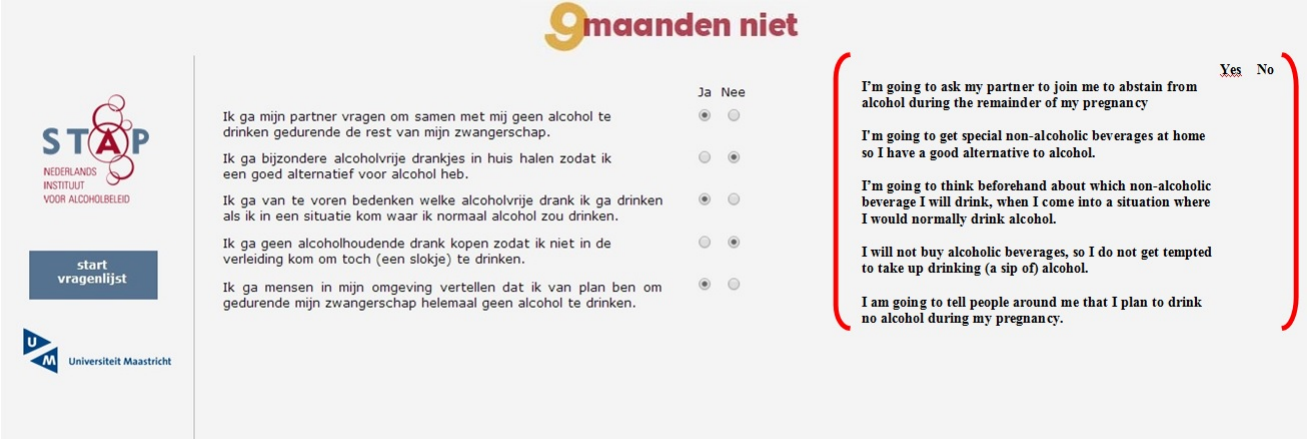
Screenshot and English translation of items regarding action plans to abstain from prenatal alcohol use.

**Figure 2:**
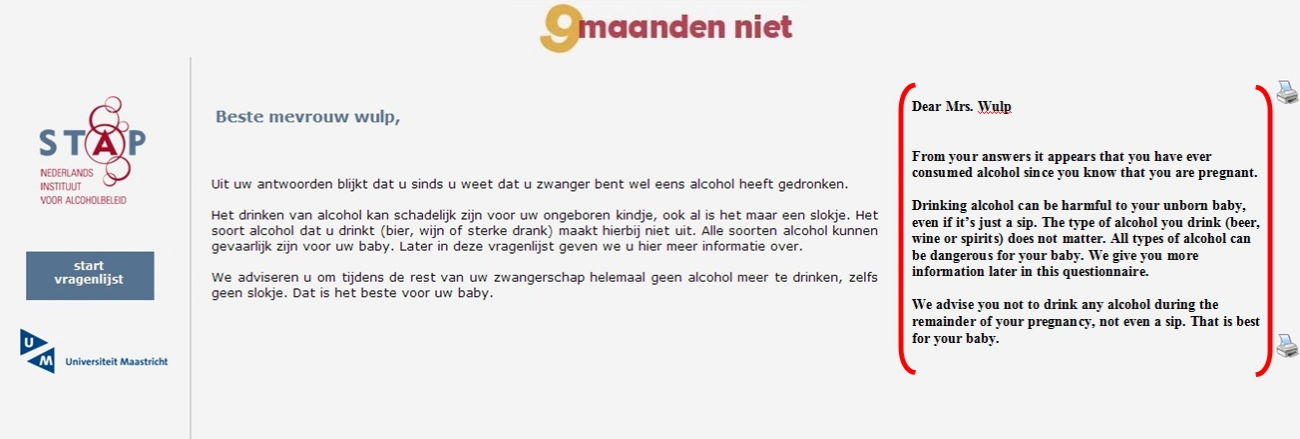
Screenshot and English translation of personal advice regarding prenatal alcohol use.

The computer-tailored intervention was developed using Tailorbuilder software (OSE, the Netherlands), a program which is specifically designed to develop Web-based computer-tailored interventions. Respondents in the computer-tailoring group received usual care from their midwife and computer-tailored feedback via the Internet, which was iterative and item-based [35]. Feedback 1, given immediately after baseline, consisted of 4 to 5 pages (Feedback 1-computer tailoring). This feedback was tailored to several respondent characteristics assessed in the baseline questionnaire: alcohol use, knowledge, risk perception, attitude, social influence, self-efficacy, intention, and action and coping plans. Specifically, the first feedback letter contained the recommendation of complete alcohol abstinence during pregnancy and information on possible consequences of prenatal alcohol use and the associated risk factors. In addition, feedback was provided on the respondent’s risk perception of prenatal alcohol use; her attitude (perceived advantages and disadvantages toward prenatal alcohol use and alcohol abstinence; perceived social influence (not) to drink during pregnancy; self-efficacy to refrain from prenatal alcohol use in specific situations, including suggestions on how to cope with these situations; the extent to which respondents were planning to undertake specific actions (action plans) to abstain from prenatal alcohol use; and how to cope with certain difficult situations (coping plans), including the formulation of personal plans in the shape of if-then statements [36]. The second feedback letter, 6 weeks after baseline, included personalized information on the respondents’ choice of characteristics assessed with the baseline questionnaire (eg, risk perception or attitude; Feedback 2-computer tailoring). Depending on the number of characteristics chosen by the respondent, this feedback consisted of 1 or 2 pages. The third feedback letter, given immediately after T1, consisted of 3 to 4 pages of ipsative feedback tailored to changes in the respondent characteristics assessed at T1 in comparison to the baseline questionnaire (Feedback 3-computer tailoring). Feedback letters were visible on the computer screen and also sent to the respondent by email. Figure 1 shows an example of items regarding action plans to abstain from prenatal alcohol use. Figure 2 shows an example of a tailored feedback message.

#### Usual Care

Midwives in the usual care group were instructed to give routine alcohol care. In-line with national guidelines, midwives recommend complete alcohol abstinence to clients who are using alcohol in the initial consultation [37,38]. In practice, not much time is spent on this nor is it common to provide additional counseling or other information [39].

### Pretests of the Interventions

The midwives’ manual of the health-counseling intervention was pretested among 5 midwives and the computer-tailoring intervention was pretested among 5 pregnant women using alcohol. The pretests yielded information about unclear questions and formulations in the manual and in the computer-tailoring intervention, which was used to improve the texts in the final versions of the health-counseling manual and computer-tailoring intervention.

### Measures

#### Baseline Questionnaire

The baseline questionnaire required 15 minutes to complete, consisted of 92 questions, and was based on questionnaires in previous studies applying the I-Change model [40-42]. Questions assessed alcohol use in pregnancy (average alcohol use, binge drinking, and risky drinking), predisposing factors (drinking behavior before pregnancy, demographics, and smoking behavior), awareness factors (risk perception), and motivational factors (attitude, social influences, and self-efficacy).

Average alcohol consumption during pregnancy was assessed with the 5-item Dutch Quantity-Frequency-Variability (QFV) questionnaire [43]. Respondents selected the type of alcoholic drinks that they had consumed since the beginning of their pregnancy, such as beer, wine, or cocktails. Respondents were asked to indicate how many working days (Monday to Thursday) on average they had consumed this type of alcohol since the beginning of their pregnancy. Additionally, they were asked to indicate the quantity (number of sips, glasses, or bottles) they had usually consumed of this type of alcohol on these occasions. Similar questions were asked concerning alcohol consumption during weekend days (Friday to Sunday). The average number of drinking working days multiplied by the average alcohol consumption per working day plus the average number of drinking weekend days multiplied by the average alcohol consumption per weekend day comprised the average weekly alcohol consumption during pregnancy.

We assessed 2 types of hazardous prenatal drinking behavior because previous research on alcohol use in pregnancy often used either of these types [44]. First, binge drinking in pregnancy was assessed by asking respondents if they ever had 4 or more standard glasses of alcohol (consisting of 10 grams of pure alcohol) on 1 day since they knew they were pregnant (0=no; 1=yes). Second, risky drinking during pregnancy was assessed with the validated T-ACE screening tool [45] (0=not risky drinking; 1=risky drinking).

Average alcohol consumption before pregnancy was also assessed with the QFV and was calculated similarly to the average weekly alcohol consumption during pregnancy.

Demographic information regarding age, education (primary school/basic vocational school, secondary vocational school/high school degree, higher vocational school/college degree/university degree), income (0.5 or less; 0.5-2; more than 2 times net Dutch median household income), and relationship status (0=no steady partner; 1=steady partner) was assessed. Pregnancy-related questions included number of weeks pregnant, number of prior pregnancies (0, 1, 2, or more than 2 prior pregnancies), and experience with complications in a previous pregnancy (0=no; 1=yes).

Respondents’ smoking behavior (in number of cigarettes per day) during and before pregnancy was assessed (“On average, how many cigarettes do you smoke per day”).

Risk perception was operationalized with perceived likelihood that the baby would experience harm (“If I drink alcohol in pregnancy, the chance that it damages my baby is...”; 1=very low; 5=very high) and perceived severity of that harm (“If I drink alcohol in pregnancy, the severity of the damage due to that alcohol is...”; 1=not serious; 5=very serious) resulting from alcohol use during pregnancy.

Attitude concerning alcohol use in pregnancy was assessed using 12 items (–2=disagree; 2=agree). A factor analysis using varimax rotation revealed 2 components: advantages (pros) and disadvantages (cons) of not drinking alcohol in pregnancy. Pros were assessed by 6 items (α=.75), such as “If I do not drink any alcohol in pregnancy, it is better for my baby’s health.” Cons of not drinking alcohol in pregnancy were assessed by another 6 items (α=.82), such as “If I do not drink any alcohol in pregnancy, I feel more tense.”

Social support to abstain from alcohol in pregnancy was assessed with 3 items on a 5-point scale (α=.91) asking respondents whether they were supported by their partner, mother, and friends to abstain from alcohol in pregnancy, such as “My partner supports me not to drink alcohol in pregnancy” (–2=totally disagree; 2=totally agree).

Self-efficacy toward alcohol abstinence in pregnancy in social situations was assessed by 6 items on a 5-point scale (α=.90), such as “How easy it is for you to abstain from alcohol when your partner drinks alcohol” (–2=very difficult; 2=very easy).

#### Follow-Up Questionnaires

Posttest drinking behavior (“Have you had at least 1 sip of alcohol since the previous questionnaire?”; 0=no; 1=yes) and average weekly alcohol consumption since the previous questionnaire (assessed with the QFV) were assessed at T1 and at T2.

### Analyses

The respondents who had had a miscarriage since the baseline were excluded from the analyses. The other respondents who did not complete the posttest questionnaire remained in the dataset and were considered as missing at random (MAR). Because respondents were nested in midwifery practices, all analyses were conducted using a mixed model analysis (SPSS v19).

To test whether conditions differed with regard to dropout, logistic mixed model analyses of dropout at T1 and T2 (0=no dropout; 1=dropout due to miscarriage, being unreachable, or being no longer interested to participate) were conducted with condition, age, education, steady partner, number of prior pregnancies, alcohol use before pregnancy, and smoking as independent variables.

To check for potentially confounding variables, univariate linear regressions with condition as predictor were performed and tested whether baseline characteristics of respondents differed between the 3 conditions.

In a set of multiple logistic mixed model analyses, we investigated the effect of condition in addition to the effect of covariates (concepts of the I-Change model) on posttest drinking behavior at T1 and T2 (0=not drinking; 1=still drinking). Significant interactions of covariates with condition were detected in a set of multiple logistic regression analyses conducted in a top-down procedure in which the least significant interaction, with *P*>.05, was omitted from a subsequent analysis. Significant main effects of covariates were also detected in a set of multiple logistic regression analyses conducted in a top-down procedure in which the least significant main effect, with *P*>.05, was omitted from a subsequent analysis. If there were no significant interaction effects with condition, we conducted a final multiple logistic regression with condition and the significant main effects of covariates and drinking behavior at T1 and T2 as outcome variable. If there were significant interaction effects with condition, we probed the interaction to understand the role of condition. Following Hayes and Matthes [46], we used the pick-a-point approach and tested whether condition was significant at 3 points on the moderator variable (1 standard deviation below average, average, and 1 standard deviation above average).

For respondents who were still drinking alcohol at T1 and T2, we tested the effect of condition in addition to the effect of covariates on the reduction of alcohol use. We performed similar sets of analyses as described previously using multiple linear mixed model analysis to assess the effect of condition in addition to the effect of confounding and moderating variables on average weekly alcohol consumption. Because of a right-skewed distribution (relatively few respondents had a high average weekly alcohol consumption), a transformation by the natural logarithm was applied to the average weekly alcohol consumption at T1 and T2.

Finally, sensitivity analyses were conducted to test the robustness of the MAR assumption for the first hypothesis. These sensitivity analyses comprised the elaboration of 3 scenarios. First, all missing values were considered as still drinking alcohol; second, all missing values were considered as having stopped drinking alcohol. The third scenario entailed that women in the health-counseling condition who had quit alcohol were as likely as those who had not quit alcohol to return the follow-up questionnaire (eg, because of their connection with their midwife) whereas women in the computer tailoring and usual care conditions who had quit alcohol were twice as likely to return the follow-up questionnaire than those who had not quit alcohol (eg, because they wanted the researchers to know they had been successful). The robustness of the MAR assumption is supported when outcomes of these scenarios (including significant covariates) are similar to the outcomes of the analyses without the imputation of the missing values [47].

## Results

### Recruitment Results

**Figure 3:**
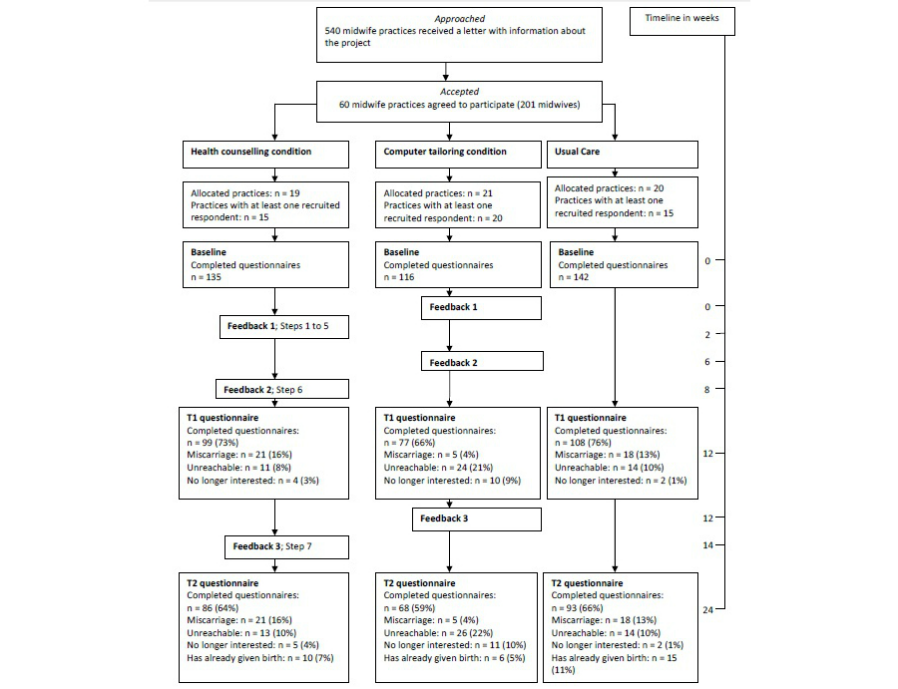
Flowchart of cluster randomized trial testing the effectiveness of health counseling and computer tailoring compared to usual care.

The baseline questionnaire was completed by 393 respondents. In total, 135 respondents were assigned to the health-counseling condition, 116 respondents to the computer-tailoring condition, and 142 respondents to the usual care condition (Figure 3). These numbers varied slightly per condition because midwives in the 3 conditions yielded a slightly different number of participating women.

### Selective Dropout

A total of 99 of 135 health-counseling respondents (73.3%), 77 of 116 computer-tailoring respondents (66.4%), and 108 of 142 usual care respondents (76.1%) completed T1. Multilevel logistic regression analysis with dropout (no/yes) at T1 as outcome variable and condition, age, education, steady partner, number of prior pregnancies, alcohol use before pregnancy, and smoking as covariates showed a significant fixed effect for having a steady partner. Respondents without a steady partner (OR 0.497, 95% CI 0.305-0.809; *P*=.005) were significantly less likely to drop out at T1. Condition and random effects were not significant.

A total of 86 of 135 health-counseling respondents (63.7%), 68 of 116 computer-tailoring respondents (58.6%), and 93 of 142 usual care respondents (65.5%) completed the T2 questionnaire. Multilevel logistic regression analysis with dropout (no/yes) at T2 as outcome variable did not show a significant effect for condition or any other factor.

### Sample Characteristics

Analyses on sample characteristics were conducted on the baseline characteristics of all respondents except 44 respondents with a miscarriage (114 health-counseling respondents, 111 computer-tailoring respondents, 124 usual care respondents; see Table 1). This sample had a mean age of 32.6 (SD 4.20) years. Most women were highly educated and had a medium income. The respondents were, on average, nearly 8 weeks pregnant, had been drinking almost 6 standard drinks of alcohol per week prior to pregnancy, and drank 1 standard drink of alcohol per week during pregnancy.

Multilevel analyses with sample characteristics as outcome variables and condition as predictor showed that computer-tailoring respondents drank significantly less alcohol before pregnancy compared to usual care respondents, and that health-counseling and computer-tailoring respondents smoked cigarettes more often compared to usual care respondents. Thus, alcohol use before pregnancy and smoking were considered potentially confounding variables in subsequent analyses. At the level of midwifery practices, respondents differed significantly with regard to educational level, income, and number of weeks of pregnancy indicating the necessity of a multilevel approach in the subsequent analyses.

**Table 1:** Baseline sample characteristics of Dutch pregnant women using alcohol (N=349).

Characteristic		Overall sample (N=349)	Health counseling (n=114)	Computer tailoring (n=111)	Usual care (n=124)	Condition effect, *P*	Random effect of midwifery practices, *P*
Age (years), mean (SD)		32.56 (4.20)	31.75 (4.37)	32.31 (4.22)	33.53 (3.85)	.17	.07
**Educational level, n (%)**						.15	.02
	Low	9 (2.6)	5 (4.5)	1 (0.9)	3 (2.4)		
	Medium	108 (31.2)	47 (42.0)	41 (36.9)	20 (16.3)		
	High	229 (66.2)	60 (53.6)	69 (62.2)	100 (81.3)		
**Income, n (%)**						.93	.03
	Low	35 (11.3)	14 (13.9)	9 (9.0)	12 (11.0)		
	Medium	170 (54.8)	56 (55.4)	62 (62.0)	52 (47.7)		
	High	105 (33.9)	31 (30.7)	29 (29.0)	45 (41.3)		
Steady partner, n (%)		198 (56.7)	73 (64.0)	66 (59.5)	59 (47.6)	.17	.33
**Number of prior pregnancies, n (%)**						.33	.14
	0	150 (43.0)	51 (44.7)	37 (33.3)	62 (50.0)		
	1	113 (32.4)	30 (26.3)	44 (39.6)	39 (31.5)		
	2	56 (16.0)	23 (20.2)	16 (14.4)	17 (13.7)		
	>2	30 (8.6)	10 (8.8)	14 (12.6)	6 (4.8)		
Number of weeks pregnant, mean (SD)		7.87 (1.96)	7.96 (1.81)	7.73 (2.06)	7.92 (1.99)	.72	.02
Experienced complications in previous pregnancy, n (%)		76 (22.4)	23 (20.9)	26 (23.9)	27 (22.3)	.87	.92
Standard alcohol drinks per week during pregnancy, mean (SD)		1.13 (2.87)	1.44 (3.33)	1.21 (3.14)	0.76 (2.02)	.23	.72
Binge drinkers during pregnancy,^a^ n (%)		4 (1.2)	3 (2.7)	0 (0)	1 (0.8)	.17	—
Risky drinkers (T-ACE positive), n (%)		198 (57.4)	73 (64.6)	55 (50.9)	70 (56.5)	.13	.93
Standard alcohol drinks per week before pregnancy, mean (SD)		5.83 (7.35)	5.61 (8.88)	4.53 (4.61)^b^	7.18 (7.59)	.06	.62
Smokes in pregnancy,^a^ n (%)		69 (20.2)	30 (27.0)^b^	25 (23.4)^b^	14 (11.3)	.01	—

^a^ Single-level analyses were conducted on the characteristics binge drinking and smoking during pregnancy because in the multilevel analyses, the estimates of the variances of the random effects were 0 and the Hessian matrices were not positive definite.

^b^Indicates significant difference compared to usual care.

### Drinking Behavior at T1 and T2

Our first hypothesis stated that women receiving health counseling or computer tailoring would be more likely to stop using alcohol in pregnancy compared to women receiving usual care at T1 and at T2. The results at T1 did not support our hypothesis. At T1, 64 of 99 health-counseling respondents (65%), 54 of 77 computer-tailoring respondents (70%), and 49 of 108 usual care respondents (45.4%) had refrained from alcohol. These differences were not significant (*P*=.79 for health counseling vs usual care; *P*=.15 for computer tailoring vs usual care; *P*=.23 for health counseling vs computer tailoring).

At T2, 62 of 86 health-counseling respondents (72%), 53 of 68 computer-tailoring respondents (78%), and 51 of 93 of the usual care respondents (55%) had refrained from alcohol. Table 2 presents the final model of the multilevel multiple logistic regression analyses with drinking behavior at T2 as outcome variable. The effects of the covariates (ie, alcohol use before pregnancy, smoking, age, education, perceived likelihood and perceived severity of risk due to prenatal alcohol use, pros and cons of not drinking alcohol in pregnancy, social support to abstain from alcohol in pregnancy, and social self-efficacy) were tested for significance and, if not significant, removed from the analysis model. The final analysis model showed that computer-tailoring respondents had refrained from alcohol significantly more often compared to usual care respondents, supporting our first hypothesis. However, the difference between health-counseling and usual care respondents was not significant (*P*=.26). Moreover, the difference between computer-tailoring and health-counseling respondents was not significant (*P*=.32).

**Table 2:** Final model of the multilevel multiple logistic regression analysis concerning drinking behavior at T2 (N=241).^a^

Fixed effects		Estimated variance	B	SE	OR	95% CI	*P*
	Health counseling^b^		0.52	0.46	1.68	0.68, 4.18	.26
	Computer tailoring^b^		1.02	0.49	2.77	1.05, 7.34	.04
	Age		–0.11	0.05	0.89	0.82, 0.98	.01
	Perceived likelihood		0.48	0.16	1.61	1.18, 2.19	.003
	Self-efficacy		0.53	0.18	1.69	1.19, 2.41	.004
Random effect		0.40		0.36			.13

^a^ 6 respondents were lost because they had not filled in the questions about self-efficacy in social situations.

^b^ Usual care is the reference category.

### Average Weekly Alcohol Consumption at T1 and T2

Our second hypothesis stated that women who continued their alcohol use would be more successful in reducing their alcohol consumption after receiving health counseling or computer tailoring at T1 and at T2. This hypothesis was not supported at T1. Only considering respondents who had not stopped drinking alcohol (n=35 in health counseling; n=23 in computer tailoring; n=59 in usual care), health-counseling respondents drank on average 0.56 standard drinks of alcohol per week (SD 0.91), computer-tailoring respondents drank 0.27 units (SD 0.17), and usual care respondents drank 0.51 units (SD 0.54). These differences were not significant (*P*=.58 for health counseling vs usual care; *P*=.23 for computer tailoring vs usual care; *P*=.49 for health counseling vs computer tailoring).

Our second hypothesis was partially supported at T2. Table 3 summarizes the results of the multilevel multiple linear regression analyses with average weekly alcohol consumption at T2 for those respondents who had not stopped drinking alcohol (n=23 in health counseling; n=15 in computer tailoring; n=41 in usual care). Health-counseling respondents drank on average 0.77 standard drinks of alcohol per week (SD 1.36), computer-tailoring respondents drank 0.35 units (SD 0.31), and usual care respondents drank 0.48 units (SD 0.54). Due to the significant interaction effects of computer tailoring with alcohol use before pregnancy and computer tailoring with social support, the main effect of computer tailoring could not be interpreted (see Table 3). To understand for which persons computer tailoring had a significant effect on the reduction of their alcohol use and for which persons computer tailoring was not significant, we probed the interactions by means of the pick-a-point approach. This showed that computer tailoring significantly reduced the alcohol use at T2 compared to usual care among respondents who had an average (*P*=.007) or 1 standard deviation below the average of alcohol use before pregnancy (*P*<.001), but not among respondents 1 standard deviation above the average of alcohol use before pregnancy (*P*=.57). In addition, computer tailoring significantly reduced alcohol use at T2 compared to usual care among respondents average (*P*=.03) or 1 standard deviation below the average of social support (*P*=.002), but not among respondents 1 standard deviation above the average of social support (*P*=.87). The analyses additionally showed that health counseling was not significant.

**Table 3:** Final model of the multilevel multiple linear regression analysis with the natural logarithm of average alcohol consumption at T2 as outcome variable among alcohol users only (N=73).^a^

Fixed effects		Estimated variance	B	SE	95% CI	*P*
	Health counseling^b^		–1.11	0.92	–2.94, 0.72	.23
	Computer tailoring^b^		6.41	1.75	2.92, 9.90	<.001
	Not smoking^c^		–1.23	0.40	–2.03, –0.43	.003
	Alcohol use before pregnancy		0.00	0.03	–0.06, 0.06	.95
	Social support		0.16	0.16	–0.16, 0.47	.34
	Health counseling^b^ * alcohol use before pregnancy		–0.05	0.05	–0.15, 0.05	.32
	Computer tailoring^b^ * alcohol use before pregnancy		–0.43	0.12	–0.67, –0.18	.001
	Health counseling^b^ * social support		0.39	0.24	–0.08, 0.87	.10
	Computer tailoring^b^ * social support		–1.38	0.35	–2.08, –0.67	<.001
Random effect		0		0		

^a^ 2 respondents were not included because they had not reported the amount of alcohol use; 6 respondents were lost because they had not filled in the question about social support.

^b^ Usual care is the reference category.

^c^ Smoking is the reference category.

### Sensitivity Analyses for Missing-at-Random Assumption of Posttest Drinking Behavior (No/Yes)

We conducted sensitivity analyses for drinking behavior at T2 because with this outcome variable computer tailoring differed significantly from usual care. Scenario 1 entailed that all missing values were replaced with 1 (still drinking alcohol at T2). In scenario 1, 62 of 135 health-counseling respondents (45.9%), 53 of 116 computer-tailoring respondents (45.7%), and 51 of 142 usual care respondents (35.9%) refrained from alcohol. A multiple logistic regression analysis with drinking behavior 6 months after baseline according to scenario 1 as outcome variable showed that more computer-tailoring respondents refrained from alcohol than usual care respondents (*P*=.06). The difference between health counseling and usual care was not significant (*P*=.46).

Scenario 2 entailed that all missing values were replaced by 0 (not drinking alcohol at T2). In scenario 2, 111 of 135 health-counseling respondents (82.2%), 101 of 116 computer-tailoring respondents (87.1%), and 100 of 142 usual care respondents (70.4%) refrained from alcohol. A multiple logistic regression analysis with drinking behavior 6 months after baseline according to scenario 2 as outcome variable showed that significantly more computer-tailoring respondents refrained from alcohol than usual care respondents (*P*=.04). The difference between health counseling and usual care was not significant (*P*=.35).

In scenario 3, 36 of 49 missing values in the health-counseling condition (73%) were randomly replaced by 0 (not drinking alcohol at T2) and 13 of 49 missing values (27%) by 1 (still drinking alcohol at T2); in the computer-tailoring condition, 19 of 48 missing values (40%) were randomly replaced by 0 and 29 of 48 missing values (60%) by 1; in the usual care condition, 14 of 49 missing values (29%) were randomly replaced by 0 and 35 of 49 missing values (71%) by 1. A multiple logistic regression analysis with drinking behavior 6 months after baseline according to scenario 3 as outcome variable showed that significantly more computer-tailoring respondents (72/116, 62.1%) and health-counseling respondents (98/135, 72.6%) refrained from alcohol than usual care respondents (65/142, 45.8%; *P*=.04 and *P*=.01, respectively).

## Discussion

The goal of this study was to test the effectiveness of 2 different brief interventions to reduce prenatal alcohol use, a health-counseling and a computer-tailored intervention, in comparison with usual care. We hypothesized that women receiving a newly developed health counseling or computer-tailored intervention were more likely to stop (hypothesis 1) and reduce (hypothesis 2) their prenatal alcohol use compared to women receiving usual care. This effect study showed that after 6 months and 3 feedback letters, the computer-tailoring program was effective in stopping prenatal alcohol use and in reducing it under certain conditions compared to usual care; the health-counseling protocol was not.

The ineffectiveness of the newly developed health-counseling protocol was inconsistent with the significant effects of health-counseling interventions in the related field of smoking cessation in pregnancy [29,48,49]. One shortcoming of this effectiveness study was the lack of statistical power. The power was planned to be .80 but turned out to be approximately .50 due to a larger intraclass correlation and a higher percentage of usual care participants who continued drinking than estimated beforehand. Although this amount of power was sufficient to show a significant effect of the computer-tailoring intervention at 6 months after baseline, the 20% difference between health-counseling and usual care respondents who stopped drinking alcohol at T1 and the 17% difference at T2 were not significant. It is unclear whether health counseling would have been found effective with more statistical power.

A second shortcoming of this study was the suboptimal implementation of the health-counseling intervention by the midwives. Our process evaluation showed that the health-counseling midwives gave counseling less extensively than they were trained [50]. For example, the majority of midwives did not offer the second and third counseling sessions because they thought their clients did not need or like to receive this successive counseling. Also, in the related field of smoking cessation in pregnancy, results were ineffective when health professionals were found to lack skills to implement their tasks as intended [29,51]. A review on the barriers and facilitators of the effective implementation of brief interventions for alcohol misuse does show that effective implementation requires adequate training in which practitioners obtain sufficient confidence and knowledge to address drinking behavior without being worried to upset patients [52].

Both shortcomings imply that the ineffectiveness of the health-counseling intervention may not be simply due to an unsuccessful protocol. Perhaps the health-counseling protocol would have led to significant effects on the reduction of prenatal alcohol use with a higher amount of power and a better implementation. Future researchers testing an intervention to reduce prenatal alcohol use are recommended to take these issues into consideration (eg, [53]).

This is the first study showing that computer tailoring is effective in reducing prenatal alcohol use. The presently reported effect is in-line with previous studies showing how computer tailoring can effectively change health-related behaviors, such as smoking [40], vegetable and fruit intake [23], and alcohol use [24]. This computer-tailoring intervention is a promising method to reduce prenatal alcohol use. The high percentage of pregnant women using alcohol in the Netherlands [4] shows that alternatives to usual care are needed. Previous research has shown that pregnant women are reluctant to disclose their alcohol use to health professionals (eg, [39]). Because computer tailoring preserves a person’s anonymity [54], computer tailoring may be an attractive intervention for these women. Moreover, the implementation of computer tailoring is not affected by barriers to the effective implementation of health counseling interventions, such as lack of resources, training, and support from management, as well as workload of practitioners providing health counseling [52]. Finally, previous research has shown that computer tailoring can be cheaper than a health-counseling intervention [55,56]; therefore, it may be a cost-effective method to decrease prenatal alcohol use, although additional research is needed to support this supposition.

A major strength of the present study was the use of a theoretical framework, which has been previously used in interventions for a variety of health behaviors (eg, [22,29,57]). In addition, both interventions used 3 feedback moments. Previous research on computer tailoring has shown that multiple feedback moments are likely to be more effective than a single feedback moment [58-60]. More research is needed to explore the optimal number of feedback moments for both computer tailoring and for health-counseling interventions. A limitation of the present study is the high percentage of dropout of respondents, especially in the computer-tailoring condition. Nevertheless, our sensitivity analyses show that the effectiveness of the computer-tailoring intervention is robust despite this high percentage of dropout. Another potential limitation is the reliance on self-report of alcohol use. Although the QFV is considered reasonably reliable [43], the use of more objective assessments, such as urine tests, may have yielded different results. Nevertheless, self-report methods of drinking (eg, QFV, the Alcohol Timeline Followback [61]) have been used in many studies on human drinking behavior because they are inexpensive, noninvasive, and acceptable to respondents [62]. Moreover, it is likely that the potential underreporting of alcohol use has occurred to an equal extent in the experimental and control conditions, upholding the effectiveness of computer tailoring. Finally, it was not possible to compare the effectiveness of computer tailoring with health counseling due to various differences in the set-ups of the interventions, including the anonymity of the respondents and the timing of the feedback. Only when the set-ups of the interventions are identical, future research will be able to compare the effectiveness of computer tailoring with health counseling.

To conclude, this research tested the effectiveness of 2 newly developed interventions to reduce prenatal alcohol use. Despite previous studies showing effects of health counseling in reducing prenatal alcohol use, our health-counseling intervention was not effective. Future studies testing health-counseling interventions are recommended to invest more in recruitment of pregnant women and implementation by health care providers. Our computer-tailoring intervention was effective in stopping and reducing prenatal alcohol use at 6-month follow-up. A cost-effectiveness study is recommended to determine the costs and effects associated with this intervention and compare them with the costs and effects of other interventions and/or usual care. A cost-effective computer-tailoring intervention would call for a broad implementation to prevent adverse neurodevelopmental effects in children due to light or moderate alcohol use.

## Multimedia Appendix 1

CONSORT-EHEALTH checklist V1.6.2 [63].

## Abbreviations

ICC: intraclass correlation coefficient
MAR: missing at random
QFV: Quantity-Frequency-Variability
