# Supplementary material for: Reducing Alcohol Use During Pregnancy Via Health Counseling by Midwives and Internet-Based Computer-Tailored Feedback: A Cluster Randomized Trial
Source: J Med Internet Res. 2014 Dec 5;16(12):e274. doi: 10.2196/jmir.3493 (PMC4275508; doi:10.2196/jmir.3493)
Supplement: Supplementary file 1 [file jmir_v16i12e274_app1.pdf]

CONSORT-EHEALTH Checklist V1.6.2 Report

(based on CONSORT-EHEALTH V1.6), available at [http://tinyurl.com/consort-ehealth-v1-6].

3493

Date completed

4/27/2014 10:40:08

by

Nickie van der Wulp

Reducing prenatal alcohol use via health counseling by midwives and Internet-based computer tailored feedback: A cluster randomized trial

TITLE

1a-i) Identify the mode of delivery in the title

"health counseling by midwives and Internet-based computer tailored feedback"

1a-ii) Non-web-based components or important co-interventions in title

See 1a-i

1a-iii) Primary condition or target group in the title

"Reducing prenatal alcohol use" indicates that target group consits of pregnant women using alcohol.

ABSTRACT

1b-i) Key features/functionalities/components of the intervention and comparator in the METHODS section of the ABSTRACT

"HC respondents received counseling from their midwife according to a HC protocol, which consisted of seven steps, addressed in three feedback sessions. CT respondents received usual care from their midwife and three CT feedback letters via the Internet. UC respondents received routine alcohol care from their midwife."

1b-ii) Level of human involvement in the METHODS section of the ABSTRACT

"HC respondents received counseling from their midwife according to a HC protocol, which consisted of seven steps, addressed in three feedback sessions. CT respondents received usual care from their midwife and three CT feedback letters via the Internet. UC respondents received routine alcohol care from their midwife."

1b-iii) Open vs. closed, web-based (self-assessment) vs. face-to-face assessments in the METHODS section of the ABSTRACT

"Sixty Dutch midwifery practices, randomly assigned to one of three conditions, recruited 135 HC, 116 CT and 142 Usual Care (UC) respondents from February to September 2011"

1b-iv) RESULTS section in abstract must contain use data

"Multilevel multivariate logistic analyses showed that CT respondents more often stopped using alcohol compared to UC respondents six months after baseline (respectively 78% versus 55%; p= .04). Multilevel multivariate linear analyses showed that CT respondents (M = 0.35 units per week; SD = 0.31) with average (p = .007) or lower (p < .001) alcohol use before pregnancy or with average (p = .03) or lower (p = .002) social support more strongly reduced their alcohol use six months after baseline compared to UC respondents (M = 0.48; SD = 0.54). Six months after baseline, 72% of the HC respondents had stopped using alcohol. This 17% difference with the UC group was not significant. "

1b-v) CONCLUSIONS/DISCUSSION in abstract for negative trials

"This is the first study showing the effectiveness of CT to reduce prenatal alcohol use. The non-significant effect of HC was probably due to a statistical power problem. "

INTRODUCTION

2a-i) Problem and the type of system/solution

"The goal of this study was to test the effectiveness of two different brief interventions to reduce prenatal alcohol use: a HC and a CT intervention, in comparison with usual care (UC). In agreement with several national recommendations [3-5], our primary focus for the development of the interventions was that pregnant women who used alcohol in the beginning of their pregnancy stopped their alcohol use after having received an intervention. "

2a-ii) Scientific background, rationale: What is known about the (type of) system

"These studies suggest that HC interventions may result in increased abstinence and a reduction in prenatal alcohol consumption. However, because of the inconsistency of the results, the paucity of studies, the relatively low number of total respondents, the high risk of bias of the studies due to lacking information on allocation concealment, and the complexities of interventions, many uncertainties remain about the most optimal conditions of these interventions [7]."

"the effectiveness of CT interventions on the reduction of prenatal alcohol use has thus far not been shown."

METHODS

3a) CONSORT: Description of trial design (such as parallel, factorial) including allocation ratio

"The goal of this study was to test the effectiveness of two different brief interventions to reduce prenatal alcohol use: a HC and a CT intervention, in comparison with usual care (UC). In agreement with several national recommendations [3-5], our primary focus for the development of the interventions was that pregnant women who used alcohol in the beginning of their pregnancy stopped their alcohol use after having received an intervention. Thus, our first hypothesis was that women receiving HC or CT were more likely to stop using alcohol in pregnancy compared to women receiving UC. However, for the pregnant women unwilling or unable to completely stop their alcohol use, we aimed at reducing their alcohol use as research has shown that the risk and severity of the effects of prenatal alcohol use are dose-related [24]. Consequently, our second hypothesis is that the interventions will result in a reduction of alcohol use of pregnant women who continued their alcohol use. "

3b) CONSORT: Important changes to methods after trial commencement (such as eligibility criteria), with reasons

"During the recruitment period, it appeared that the inclusion of nine respondents per practice would be too time-consuming. We decided to enroll sixty midwifery practices in total, expecting to recruit 4-5 respondents from each practice. "

3b-i) Bug fixes, Downtimes, Content Changes

No changes made on the interventions during the trial

4a) CONSORT: Eligibility criteria for participants

"Eligibility criteria were understanding Dutch, being 18 years of age or older, being pregnant for a maximum of 12 weeks (as respondents received follow-up questionnaires until six months after baseline) and having drunk alcohol since knowing to be pregnant. "

4a-i) Computer / Internet literacy

Internet literacy is indeed an implicit eligibility criterion. It was expected that the Internet literacy would not be a problem among the Dutch pregnant women participating in this study as Internet use is very common in the Netherlands. According to Statistics Netherlands, 99% of Dutch women in the child bearing age uses Internet at least once per week.

4a-ii) Open vs. closed, web-based vs. face-to-face assessments:

"When pregnant women agreed to participate, they were asked to visit the study website before their initial consultation. "

"Midwives in the HC condition received a brief manual, explaining the HC protocol and an intervention card with questions for the clients." "The HC protocol consisted of seven steps which were addressed in three feedback sessions. "

"Respondents could choose their own username and password and had to report their email address when signing up for the study. This way we could easily remove respondents with multiple identities from further analyses. "
"Respondents in the CT group received usual care from their midwife and CT feedback via the Internet, which was iterative and item-based [31]. "

4a-iii) Information giving during recruitment

"The study website included the baseline questionnaire (T0). After providing online informed consent, eligible women gained access to the baseline questionnaire. The website also included information about the objectives of the study, the randomization procedure and the incentive of a €10 voucher when respondents completed all questionnaires and institutional affiliations (“This research is conducted by the Dutch Institute for Alcohol Policy (STAP) and Maastricht University”).

4b) CONSORT: Settings and locations where the data were collected

"They could do this where and whenever they had access to the Internet."

4b-i) Report if outcomes were (self-)assessed through online questionnaires

"Average alcohol consumption during pregnancy was assessed with the five-item Dutch Quantity-Frequency-Variability (QFV) questionnaire [39]. Respondents selected the type of alcoholic drinks that they had consumed since the beginning of their pregnancy, such as beer, wine or cocktails. Respondents were asked to indicate how many working days (Monday to Thursday) on average they had consumed this type of alcohol since the beginning of their pregnancy. Additionally, they were asked to indicate the quantity (number of sips, glasses or bottles) they had usually consumed of this type of alcohol on these occasions. Similar questions were asked concerning alcohol consumption during weekend days (Friday to Sunday). The average number of drinking working days multiplied with the average alcohol consumption per working day plus the average number of drinking weekend days multiplied with the average alcohol consumption per weekend day comprised the average weekly alcohol consumption during pregnancy."

4b-ii) Report how institutional affiliations are displayed

"The website also included information about the objectives of the study, the randomization procedure and the incentive of a €10 voucher when respondents completed all questionnaires and institutional affiliations (“This research is conducted by the Dutch Institute for Alcohol Policy (STAP) and Maastricht University”). "

5) CONSORT: Describe the interventions for each group with sufficient details to allow replication, including how and when they were actually administered

5-i) Mention names, credential, affiliations of the developers, sponsors, and owners

The HC and CT intervention were both based on the I-Change Model [25], a theoretical model incorporating concepts from several social cognitive models, such as the Transtheoretical model [26] and the Theory of Planned Behavior [27].

HC: "The materials and training were based on earlier work on tobacco and pregnancy [28]. "

CT: "The CT intervention was developed using Tailorbuilder software (OSE, the Netherlands), a program which is specifically designed to develop web-based computer-tailored interventions." and "HdV is the scientific director of Vision2Health, a collaborating company between the University of Maastricht and OSE, with the aim of offering proven effective methods in the field of health education.

5-ii) Describe the history/development process

"The midwives' manual of the HC intervention was pretested among five midwives and the CT intervention was pretested among five pregnant women using alcohol. The pre-tests yielded useful findings, for example about unclear questions and formulations both in the manual and in the CT intervention and led to an improvement of the texts in the final versions of the HC manual and CT intervention."

5-iii) Revisions and updating

The development and content of the interventions were 'frozen' during the trial.

5-iv) Quality assurance methods

All information was based on scientific papers which can be provided on request.

5-v) Ensure replicability by publishing the source code, and/or providing screenshots/screen-capture video, and/or providing flowcharts of the algorithms used

"Figure 1 shows an example of items regarding action plans to abstain from prenatal alcohol use. Figure 2 shows an example of a tailored feedback message."

5-vi) Digital preservation

"The study website (www.alcoholenzwangerschap.nl/negenmaandenniet) included the baseline questionnaire (T0)."

5-vii) Access

"Respondents could choose their own username and password and had to report their email address when signing up for the study. "

5-viii) Mode of delivery, features/functionalities/components of the intervention and comparator, and the theoretical framework

"The HC and CT intervention were both based on the I-Change Model [25], a theoretical model incorporating concepts from several social cognitive models, such as the Transtheoretical model [26] and the Theory of Planned Behavior [27]. The I-Change model distinguishes three phases of health behavior change (awareness, motivation, action) and has been used successfully for developing various health promoting interventions, for example prenatal smoking cessation [28], smoking cessation [19, 29, 30] and increasing vegetable and fruit intake and physical activity [18].

Health Counseling
Midwives in the HC condition received a brief manual, explaining the HC protocol and an intervention card with questions for the clients. On this intervention card, midwives could record the dates of the HC sessions and the clients' answers to the midwife's questions. Midwives received three hours of training on how to provide the HC counseling. This training was given either at the research institute of the first author, or at the practice of the participating midwife. The materials and training were based on earlier work on tobacco and pregnancy [28].

The HC protocol consisted of seven steps which were addressed in three feedback sessions. Feedback session 1, approximately two weeks after baseline assessment, consisted of five steps, taking approximately 10 minutes time of the initial consultation (Feedback 1 – HC). In step 1, the midwife assessed the amount and frequency of alcohol use of the pregnant woman before and during pregnancy; of her partner during pregnancy; and the pregnant woman's motivation to stop drinking alcohol. In step 2, women strongly motivated to stop alcohol consumption during pregnancy were prompted to state the advantages of quitting. Moderately or not motivated women additionally were asked to report on their perceived disadvantages of drinking during pregnancy. The midwife then advised them to stop drinking alcohol. In step 3, the barriers for successful quitting and the mobilization of social support were discussed. In step 4, a self-help guide, adapted from an intervention on smoking in pregnancy [28], and relevant websites were mentioned. The midwife stimulated the pregnant woman to develop action plans for quitting and coping with problems they might encounter when trying not to drink alcohol. If appropriate, access to alcohol addiction services was discussed. In step 5, women were asked to set a date for quitting alcohol consumption (goal setting). Feedback session 2, approximately eight weeks after baseline, consisted of step 6, which was addressed in approximately 1 minute (Feedback 2 – HC). In this step, midwives again assessed the alcohol use of the pregnant women and asked her if she needed additional support for not drinking alcohol. Feedback session 3, approximately 14 weeks after baseline, consisted of step 7, which was also addressed in approximately 1 minute (Feedback 3 – HC). In this step, midwives discussed alcohol use and its implications for breastfeeding.

Computer Tailoring
The CT intervention was developed using Tailorbuilder software (OSE, the Netherlands), a program which is specifically designed to develop web-based computer-tailored interventions. Respondents in the CT group received usual care from their midwife and CT feedback via the Internet, which was iterative and item-based [31]. Feedback 1, given immediately after baseline, consisted of four to five pages (Feedback 1 – CT). This feedback was tailored to several respondent characteristics assessed in the baseline questionnaire: alcohol use, knowledge, risk perception, attitude, social influence, self efficacy, intention, and action and coping plans. Specifically, the first feedback letter contained the recommendation of complete alcohol abstinence during pregnancy, as well as information on possible consequences of prenatal alcohol use and the associated risk factors. In addition, feedback was provided on the respondent's risk perception of prenatal alcohol use; her attitude (perceived advantages and disadvantages toward prenatal alcohol use and alcohol abstinence; perceived social influence (not) to drink during pregnancy; self-efficacy to refrain from prenatal alcohol use in specific situations, including suggestions on how to cope with these situations; the extent to which respondents were planning to undertake specific actions (action plans) to abstain from prenatal alcohol use; and, how to cope with certain difficult situations (coping plans), including the formulation of personal plans in the shape of if-then statements [32]. The second feedback letter, six weeks after baseline, included personalized information on the respondents' choice of characteristics assessed with the baseline questionnaire (for example, risk perception or attitude; Feedback 2 - CT). Depending on the number of characteristics chosen by the respondent, this feedback consisted of one or two pages. The third feedback letter, given immediately after T1, consisted of three to four pages of ipsative feedback tailored to changes in the respondent characteristics assessed at T1 in comparison to the baseline questionnaire (Feedback 3 – CT). Feedback letters were visible on the computer screen and also sent to the respondent by email. Figure 1 shows an example of items regarding action plans to abstain from prenatal alcohol use. Figure 2 shows an example of a tailored feedback message."

5-ix) Describe use parameters

HC: "The midwife then advised them to stop drinking alcohol"

CT: "Specifically, the first feedback letter contained the recommendation of complete alcohol abstinence during pregnancy, as well as information on possible consequences of prenatal alcohol use and the associated risk factors. "

5-x) Clarify the level of human involvement

HC: "The HC protocol consisted of seven steps which were addressed in three feedback sessions. Feedback session 1, approximately two weeks after baseline assessment, consisted of five steps, taking approximately 10 minutes time of the initial consultation (Feedback 1 – HC). In step 1, the midwife assessed the amount and frequency of alcohol use of the pregnant woman before and during pregnancy; of her partner during pregnancy; and the pregnant woman's motivation to stop drinking alcohol. In step 2, women strongly motivated to stop alcohol consumption during pregnancy were prompted to state the advantages of quitting. Moderately or not motivated women additionally were asked to report on their perceived disadvantages of drinking during pregnancy. The midwife then advised them to stop drinking alcohol. In step 3, the barriers for successful quitting and the mobilization of social support were discussed. In step 4, a self-help guide, adapted from an intervention on smoking in pregnancy [28], and relevant websites were mentioned. The midwife stimulated the pregnant woman to develop action plans for quitting and coping with problems they might encounter when trying not to drink alcohol. If appropriate, access to alcohol addiction services was discussed. In step 5, women were asked to set a date for quitting alcohol consumption (goal setting). Feedback session 2, approximately eight weeks after baseline, consisted of step 6, which was addressed in approximately 1 minute (Feedback 2 – HC). In this step, midwives again assessed the alcohol use of the pregnant women and asked her if she needed additional support for not drinking alcohol. Feedback session 3, approximately 14 weeks after baseline, consisted of step 7, which was also addressed in approximately 1 minute (Feedback 3 – HC). In this step, midwives discussed alcohol use and its implications for breastfeeding."

CT: "Respondents in the CT group received usual care from their midwife and CT feedback via the Internet"

Usual Care: "Midwives in the UC group were instructed to give routine alcohol care. In line with the national guidelines, midwives recommend complete alcohol abstinence to clients who are using alcohol in the initial consultation [33, 34]. In practice not much time is spent on this, nor is it common to provide additional counseling or other information [35]."

5-xi) Report any prompts/reminders used

"Three and six months after the baseline questionnaire, all participants received an invitation by email (followed by two reminders after respectively two and four weeks) for the first follow-up questionnaire (T1 and T2, respectively). Non-respondents after two reminders were contacted by telephone to collect their data. "

5-xii) Describe any co-interventions (incl. training/support)

HC: "Midwives in the HC condition received a brief manual, explaining the HC protocol and an intervention card with questions for the clients. On this intervention card, midwives could record the dates of the HC sessions and the clients' answers to the midwife's questions. Midwives received three hours of training on how to provide the HC counseling. This training was given either at the research institute of the first author, or at the practice of the participating midwife. The materials and training were based on earlier work on tobacco and pregnancy [28].

The HC protocol consisted of seven steps which were addressed in three feedback sessions. Feedback session 1, approximately two weeks after baseline assessment, consisted of five steps, taking approximately 10 minutes time of the initial consultation (Feedback 1 – HC). In step 1, the midwife assessed the amount and frequency of alcohol use of the pregnant woman before and during pregnancy; of her partner during pregnancy; and the pregnant woman's motivation to stop drinking alcohol. In step 2, women strongly motivated to stop alcohol consumption during pregnancy were prompted to state the advantages of quitting. Moderately or not motivated women additionally were asked to report on their perceived disadvantages of drinking during pregnancy. The midwife then advised them to stop drinking alcohol. In step 3, the barriers for successful quitting and the mobilization of social support were discussed. In step 4, a self-help guide, adapted from an intervention on smoking in pregnancy [28], and relevant websites were mentioned. The midwife stimulated the pregnant woman to develop action plans for quitting and coping with problems they might encounter when trying not to drink alcohol. If appropriate, access to alcohol addiction services was discussed. In step 5, women were asked to set a date for quitting alcohol consumption (goal setting). Feedback session 2, approximately eight weeks after baseline, consisted of step 6, which was addressed in approximately 1 minute (Feedback 2 – HC). In this step, midwives again assessed the alcohol use of the pregnant women and asked her if she needed additional support for not drinking alcohol. Feedback session 3, approximately 14 weeks after baseline, consisted of step 7, which was also addressed in approximately 1 minute (Feedback 3 – HC). In this step, midwives discussed alcohol use and its implications for breastfeeding."

UC: "Midwives in the UC group were instructed to give routine alcohol care. In line with the national guidelines, midwives recommend complete alcohol abstinence to clients who are using alcohol in the initial consultation [33, 34]. In practice not much time is spent on this, nor is it common to provide additional counseling or other information [35]."

6a) CONSORT: Completely defined pre-specified primary and secondary outcome measures, including how and when they were assessed

Baseline: "Average alcohol consumption during pregnancy was assessed with the five-item Dutch Quantity-Frequency-Variability (QFV) questionnaire [39]. Respondents selected the type of alcoholic drinks that they had consumed since the beginning of their pregnancy, such as beer, wine or cocktails. Respondents were asked to indicate how many working days (Monday to Thursday) on average they had consumed this type of alcohol since the beginning of their pregnancy. Additionally, they were asked to indicate the quantity (number of sips, glasses or bottles) they had usually consumed of this type of alcohol on these occasions. Similar questions were asked concerning alcohol consumption during weekend days (Friday to Sunday). The average number of drinking working days multiplied with the average alcohol consumption per working day plus the average number of drinking weekend days multiplied with the average alcohol consumption per weekend day comprised the average weekly alcohol consumption during pregnancy."

T1 and T2: "Three and six months after the baseline questionnaire, all participants received an invitation by email (followed by two reminders after respectively two and four weeks) for the first follow-up questionnaire (T1 and T2, respectively). " ... "Post-test drinking behavior (have you had at least one sip of alcohol since the previous questionnaire; 0 = no; 1 = yes) and average weekly alcohol consumption since the previous questionnaire (assessed with the QFV) were assessed at T1 and at T2. "

6a-i) Online questionnaires: describe if they were validated for online use and apply CHERRIES items to describe how the questionnaires were designed/deployed

"The baseline questionnaire required 15 minutes to complete, consisted of 92 questions and was based on questionnaires in previous studies applying the I-Change model [36-38]. Questions assessed alcohol use in pregnancy (average alcohol use, binge drinking and risky drinking), predisposing factors (drinking behavior before pregnancy, demographics, and smoking behavior), awareness factors (risk perception) and motivational factors (attitude, social influences and self-efficacy)."

6a-ii) Describe whether and how "use" (including intensity of use/dosage) was defined/measured/monitored

"Average alcohol consumption during pregnancy was assessed with the five-item Dutch Quantity-Frequency-Variability (QFV) questionnaire [39]. "

"Post-test drinking behavior (have you had at least one sip of alcohol since the previous questionnaire; 0 = no; 1 = yes) and average weekly alcohol consumption since the previous questionnaire (assessed with the QFV) were assessed at T1 and at T2."

6a-iii) Describe whether, how, and when qualitative feedback from participants was obtained

This study did not include qualitative feedback from participants.

6b) CONSORT: Any changes to trial outcomes after the trial commenced, with reasons

not applicable as the intervention did not undergo major changes after the trial commenced

7a) CONSORT: How sample size was determined

7a-i) Describe whether and how expected attrition was taken into account when calculating the sample size

"A sample size analysis (power 0.80, α = 0.05, intra class correlation of 0.01, an estimated quit rate of 40% in each of the experimental conditions versus 20% in the control condition and the estimated inclusion of 30 midwifery practices) revealed that nine respondents per practice were needed. Estimating 10% attrition over the trial period, we aimed to include 300 respondents at baseline. "

7b) CONSORT: When applicable, explanation of any interim analyses and stopping guidelines

"During the recruitment period, it appeared that the inclusion of nine respondents per practice would be too time-consuming. We decided to enroll sixty midwifery practices in total, expecting to recruit 4-5 respondents from each practice. "

8a) CONSORT: Method used to generate the random allocation sequence

"Participating practices were randomly assigned to one of the three conditions (HC, CT or UC), in order to avoid contamination, by a computer software randomization device. "

8b) CONSORT: Type of randomisation; details of any restriction (such as blocking and block size)

not relevant as the randomization process was not restricted.

9) CONSORT: Mechanism used to implement the random allocation sequence (such as sequentially numbered containers), describing any steps taken to conceal the sequence until interventions were assigned

Not applicable. Midwife practices were informed about their condition immediately after signing up for the study.

10) CONSORT: Who generated the random allocation sequence, who enrolled participants, and who assigned participants to interventions

"Participating practices were randomly assigned to one of the three conditions (HC, CT or UC), by a computer software randomization device, in order to avoid contamination. "

11a) CONSORT: Blinding - If done, who was blinded after assignment to interventions (for example, participants, care providers, those assessing outcomes) and how

11a-i) Specify who was blinded, and who wasn't

"Blinding of respondents was not possible, as they had to take notice of whether they were receiving counselling from their midwife or tailored feedback via the computer."

11a-ii) Discuss e.g., whether participants knew which intervention was the "intervention of interest" and which one was the "comparator"
Although respondents received information about the objectives of the study, the set up of the cluster randomized trial with three conditions was rather complicated. Thus, respondents receiving HC or CT feedback may have been aware that they were in one of the interventions of interest and respondents receiving usual care may have been aware that they were in the comparator condition, but we cannot be sure about their awareness.
11b) CONSORT: If relevant, description of the similarity of interventions
There is overlap between CT and Usual Care (UC):

CT: "Respondents in the CT group received usual care from their midwife and CT feedback via the Internet, which was iterative and item-based [31]. "

UC: "Midwives in the UC group were instructed to give routine alcohol care. In line with the national guidelines, midwives recommend complete alcohol abstinence to clients who are using alcohol in the initial consultation [33, 34]. In practice not much time is spent on this, nor is it common to provide additional counseling or other information [35]."
12a) CONSORT: Statistical methods used to compare groups for primary and secondary outcomes
Primary outcomes: "In a set of multivariate logistic mixed model analyses, we investigated the effect of condition, in addition to the effect of covariates (concepts of the I-Change Model), on post-test drinking behavior at T1 and T2 (0 = not drinking; 1 = still drinking). "

Secondary outcomes: "For respondents who were still drinking alcohol at T1 and T2 we tested the effect of condition, in addition to the effect of covariates, on the reduction of alcohol use. We performed similar sets of analyses as described above using multivariate linear mixed model analysis to assess the effect of condition, in addition to the effect of confounding and moderating variables, on average weekly alcohol consumption. "
12a-i) Imputation techniques to deal with attrition / missing values
"The respondents who had had a miscarriage since the baseline were excluded from the analyses. The other respondents who did not complete the post-test questionnaire remained in the dataset and were considered as missing at random (MAR). "

"sensitivity analyses were conducted to test the robustness of the MAR-assumption for hypothesis 1. These sensitivity analyses comprised the elaboration of two scenarios, that is first, all missing values were considered as still drinking alcohol; second, all missing values were considered as having stopped drinking alcohol. The robustness of the MAR-assumption is supported when outcomes of these scenario's (including significant covariates) are similar to the outcomes of the analyses without the imputation of the missing values [43]."
12b) CONSORT: Methods for additional analyses, such as subgroup analyses and adjusted analyses
"To test whether conditions differed with regard to drop out, logistic mixed model analyses of dropout at T1 and T2 (0 = no drop out; 1 = drop out due to miscarriage, being unreachable or being no longer interested to participate) were conducted with condition, age, education, steady partner, number of prior pregnancies, alcohol use before pregnancy and smoking as independent variables. "

"To check for potentially confounding variables, univariate linear regressions with condition as predictor were performed and tested whether baseline characteristics of respondents differed between the three conditions."

"Significant interactions of covariates with condition were detected in a set of multivariate logistic analyses conducted in a top down procedure, in which each time the least significant interaction, with p > .05, was omitted from a subsequent analysis. Significant main effects of covariates were also detected in a set of multivariate logistic analyses conducted in a top down procedure, in which each time the least significant main effect, with p > .05, was omitted from a subsequent analysis. If there were no significant interaction effects with condition, we conducted a final multivariate logistic regression with condition and the significant main effects of covariates and drinking behavior at T1 and T2 as outcome variable. If there were significant interaction effects with condition, we probed the interaction to understand the role of condition. Following Hayes and Matthes [42], we used the pick-a-point approach and tested whether condition was significant at three points on the moderator variable (one standard deviation below average, average and one standard deviation above average)."

RESULTS

13a) CONSORT: For each group, the numbers of participants who were randomly assigned, received intended treatment, and were analysed for the primary outcome
"To test whether conditions differed with regard to drop out, logistic mixed model analyses of dropout at T1 and T2 (0 = no drop out; 1 = drop out due to miscarriage, being unreachable or being no longer interested to participate) were conducted with condition, age, education, steady partner, number of prior pregnancies, alcohol use before pregnancy and smoking as independent variables. "

"To check for potentially confounding variables, univariate linear regressions with condition as predictor were performed and tested whether baseline characteristics of respondents differed between the three conditions.
"

"Significant interactions of covariates with condition were detected in a set of multivariate logistic analyses conducted in a top down procedure, in which each time the least significant interaction, with p > .05, was omitted from a subsequent analysis. Significant main effects of covariates were also detected in a set of multivariate logistic analyses conducted in a top down procedure, in which each time the least significant main effect, with p > .05, was omitted from a subsequent analysis. "
13b) CONSORT: For each group, losses and exclusions after randomisation, together with reasons
Figure 3 is the consort flow diagram.
13b-i) Attrition diagram
Not added as consort flow diagram present all information on attrition that is available.
14a) CONSORT: Dates defining the periods of recruitment and follow-up
"Respondents were recruited from February to September 2011."

"Three and six months after the baseline questionnaire, all participants received an invitation by email (followed by two reminders after respectively two and four weeks) for the first follow-up questionnaire (T1 and T2, respectively)."
14a-i) Indicate if critical "secular events" fell into the study period
not relevant, as there were no critical 'secular events' during the study period.
14b) CONSORT: Why the trial ended or was stopped (early)
Not relevant. We stopped recruiting when we had reached 300 respondents at baseline, just as we had planned.
15) CONSORT: A table showing baseline demographic and clinical characteristics for each group
Table 1 presents sample characteristics for each group
15-i) Report demographics associated with digital divide issues
In Table 1, data on age, education, and income are presented.

16a) CONSORT: For each group, number of participants (denominator) included in each analysis and whether the analysis was by original assigned groups

16-i) Report multiple "denominators" and provide definitions

"A total of 99 HC respondents (73.3% of the baseline HC respondents); 77 CT respondents (66.4% of the baseline CT respondents) and 108 UC respondents (76.1% of the baseline UC respondents) completed T1.

A total of 86 HC respondents (63.7% of the HC baseline respondents); 68 CT respondents (58.6% of the CT baseline respondents) and 93 UC respondents (65.5% of the UC baseline respondents) completed the T2 questionnaire. "

16-ii) Primary analysis should be intent-to-treat

"The respondents who had had a miscarriage since the baseline were excluded from the analyses. The other respondents who did not complete the post-test questionnaire remained in the dataset and were considered as missing at random (MAR). "

"sensitivity analyses were conducted to test the robustness of the MAR-assumption for hypothesis 1. These sensitivity analyses comprised the elaboration of two scenarios, that is first, all missing values were considered as still drinking alcohol; second, all missing values were considered as having stopped drinking alcohol. The robustness of the MAR-assumption is supported when outcomes of these scenario's (including significant covariates) are similar to the outcomes of the analyses without the imputation of the missing values [43]."

17a) CONSORT: For each primary and secondary outcome, results for each group, and the estimated effect size and its precision (such as 95% confidence interval)

Yes, see Tables 2 and 3.

17a-i) Presentation of process outcomes such as metrics of use and intensity of use

Not relevant for this type of intervention.

17b) CONSORT: For binary outcomes, presentation of both absolute and relative effect sizes is recommended

Absolute effect sizes in text: "These analyses showed that CT respondents (78%) had significantly more often refrained from alcohol compared to UC respondents (55%; $p = .04$), supporting our first hypothesis. The difference between HC (72%) and UC respondents ($p = .26$) was, however, not significant. Moreover, the difference between CT and HC respondents ($p = .32$) was not significant either. "

Relative effect sizes in Table 2

18) CONSORT: Results of any other analyses performed, including subgroup analyses and adjusted analyses, distinguishing pre-specified from exploratory

Significant main and interaction effects of covariates were assessed as well, see Tables 2 and 3.

18-i) Subgroup analysis of comparing only users

"In our second hypothesis we stated that women who continued their alcohol use would be more successful in reducing their alcohol consumption after receiving HC or CT at T1 as well as at T2. "

19) CONSORT: All important harms or unintended effects in each group

Not applicable: there were no important harms or unintended effects.

19-i) Include privacy breaches, technical problems

There were no privacy breaches, technical problems or other unexpected/unintended incidents.

19-ii) Include qualitative feedback from participants or observations from staff/researchers

Not available.

DISCUSSION

20) CONSORT: Trial limitations, addressing sources of potential bias, imprecision, multiplicity of analyses

20-i) Typical limitations in ehealth trials

"A limitation of the present study is the high percentage of drop out of respondents, especially in the CT condition. Nevertheless, our sensitivity analyses show that the effectiveness of the CT intervention is robust despite this high percentage of drop out. Another potential limitation is the reliance on self report of alcohol use. Although the QFV is considered reasonably reliable [39], the use of more objective assessments, as for example urine tests, may have yielded different results. Nevertheless, self-report methods of drinking (QFV, the alcohol timeline follow back, TLFB; [54], etc) have been used in many studies on human drinking behavior, because they are inexpensive, non-invasive and acceptable to respondents [55]. Moreover, it is likely that the potential underreporting of alcohol use has occurred to an equal extent in the experimental and control conditions, upholding the effectiveness of CT. Finally, it was not possible to compare the effectiveness of CT, due to various differences in the set-ups of the intervention, including for example the anonymity of the respondents and the timing of the feedback. Only when the set-ups of the interventions are identical, future research is able to compare the effectiveness of CT with HC. "

21) CONSORT: Generalisability (external validity, applicability) of the trial findings

21-i) Generalizability to other populations

"The presently reported effect is in line with previous studies showing how CT can effectively change health related behaviors, like smoking [36], vegetable and fruit intake [20] and alcohol use [21]. This CT intervention is a promising method to reduce prenatal alcohol use. The high percentage of pregnant women using alcohol in the Netherlands [4] shows that alternatives to usual care are needed. Previous research has shown that pregnant women are reluctant to disclose their alcohol use to health professionals (e.g. [35]). As CT preserves a person's anonymity [49], CT may be an attractive intervention for these women. Finally, previous research has shown that CT can be cheaper than a health counseling intervention [50, 51] and may therefore be a cost-effective method to decrease prenatal alcohol use, although additional research is needed to support this supposition."

21-ii) Discuss if there were elements in the RCT that would be different in a routine application setting

Not applicable: CT intervention is expected to be the same in a routine application setting.

22) CONSORT: Interpretation consistent with results, balancing benefits and harms, and considering other relevant evidence

22-i) Restate study questions and summarize the answers suggested by the data, starting with primary outcomes and process outcomes (use)

"The goal of this study was to test the effectiveness of two different brief interventions to reduce prenatal alcohol use: a HC and a CT intervention, in comparison with usual care (UC). We hypothesized that women receiving a newly developed HC or CT intervention were more likely to stop (hypothesis 1) and reduce (hypothesis 2) their prenatal alcohol use compared to women receiving UC. This effect study showed that after six months and three feedback letters, the CT program was effective in stopping prenatal alcohol use and in reducing it under certain conditions compared to UC; the HC protocol was not."

22-ii) Highlight unanswered new questions, suggest future research

"Both explanations imply that the ineffectiveness of the HC intervention may not be simply due to an unsuccessful protocol. In contrast, the HC protocol might have led to significant effects on the reduction of prenatal alcohol use with a higher amount of power and a better implementation. Future researchers developing an intervention to reduce prenatal alcohol use are recommended to take these issues into consideration (e.g. [48])."

"previous research has shown that CT can be cheaper than a health counseling intervention [50, 51] and may therefore be a cost-effective method to decrease prenatal alcohol use, although additional research is needed to support this supposition."

"A thorough evaluation of the implementation during the study is recommended for future studies (e.g. [48]) to warrant that the intervention will be implemented as intended. "

"Finally, it was not possible to compare the effectiveness of CT, due to various differences in the set-ups of the intervention, including for example the anonymity of the respondents and the timing of the feedback. Only when the set-ups of the interventions are identical, future research is able to compare the effectiveness of CT with HC. "

Other information

23) CONSORT: Registration number and name of trial registry

"The study was approved by the Medical Ethics Committee of Maastricht University and the University Hospital Maastricht (MEC 09-3-070) and is registered with the Dutch Trial Register (NTR2058). "

24) CONSORT: Where the full trial protocol can be accessed, if available

"The study website (www.alcoholenzwangerschap.nl/negenmaandenniet) ..."

25) CONSORT: Sources of funding and other support (such as supply of drugs), role of funders

"This study was funded by a grant of the Dutch Organization for Health Research and Development (ZonMW). "

X26-i) Comment on ethics committee approval

"The study was approved by the Medical Ethics Committee of Maastricht University and the University Hospital Maastricht (MEC 09-3-070) and is registered with the Dutch Trial Register (NTR2058). "

x26-ii) Outline informed consent procedures

"After providing online informed consent, eligible women gained access to the baseline questionnaire."

X26-iii) Safety and security procedures

Safety and security procedures were necessary to get approval of the Medical Ethics Committee of Maastricht University and the University Hospital Maastricht (MEC 09-3-070).

X27-i) State the relation of the study team towards the system being evaluated

"HdV is the scientific director of Vision2Health, a collaborating company between the University of Maastricht and OSE, with the aim of offering proven effective methods in the field of health education.
NvdW, CH, KE, MC and WvD declare that they have no conflict of interest."
